# SCOUT^®^ Radar Reflector for Nonpalpable Breast Lesion Localization: Clinical Outcomes from a Single-Center Experience

**DOI:** 10.3390/cancers17233816

**Published:** 2025-11-28

**Authors:** Julieta Puente-Monserrat, Ernesto Muñoz-Sornosa, Vicente López-Flor, Marcos Adrianzén-Vargas, Dixie Huntley-Pascual, Georgy Kadzhaya-Khlystov, Diego Soriano-Mena, Elvira Buch-Villa

**Affiliations:** 1 Breast Unit, Department of General and Digestive Surgery, Hospital Clínico Universitario of Valencia, 46010 Valencia, Spain; 2 Biomedical Research Institute, INCLIVA, 46010 Valencia, Spain; 3 Department of Surgery, University of Valencia, 46010 Valencia, Spain; 4 Department of Radiology, Hospital Clínico Universitario, 46010 Valencia, Spain

**Keywords:** breast cancer, negative margins, non-palpable lesions, SCOUT, conservative breast surgery, radar localization

## Abstract

Background: Preoperative localization of non-palpable breast lesions is critical for accurate resection and margin control in breast-conserving surgery. Traditional methods, such as wire or radioguided localization, have limitations in terms of logistics, patient comfort, and procedural flexibility. SCOUT^®^ is a wireless, radar-based alternative that may improve surgical precision and workflow. This study aimed to evaluate the clinical performance of the SCOUT^®^ in the localization of non-palpable breast and axillary lesions, including detection success, margin status, reoperation rates, and device-related events. Methods: We conducted a retrospective, single-centre observational study including 427 patients who underwent breast-conserving surgery after preoperative localization using the SCOUT^®^ between January 2023 and May 2024 at a tertiary academic hospital. Variables included lesion type, location, neoadjuvant treatment, device detection, seed deactivation, MRI interference, margin status, and reoperation rate. Results: The mean age was 58 ± 12.7 years, with malignant pathology in 88.5% of cases. SCOUT^®^ achieved a 100% detection rate in axillary localizations and 98.1% in breast lesions. Seed deactivation occurred in 1.2% of cases, all successfully managed intraoperatively. MRI artefacts were observed in 1.6% of patients, without diagnostic interference. Positive margins were reported in 8.3% of cases, representing an improvement compared with the institution’s historic 12% rate, with 5.9% requiring reoperation. Carcinoma in situ showed the highest rate of positive margins, at 26%. Conclusions: SCOUT^®^ was associated with high detection rates, a low incidence of device-related events, and favourable margin outcomes, supporting its reliability for the localization of non-palpable breast lesions.

## Simple Summary

Early breast cancer is often detected when lesions are non-palpable. Surgeons must locate these lesions before removing them, but traditional methods can cause discomfort and may complicate the operation. SCOUT^®^ is a small device that guides the surgeon using radar signals. In our study, SCOUT^®^ helped to accurately locate the lesion, achieve free margins, and avoid repeat surgeries. These results suggest that this technique can improve surgical outcomes in breast-conserving procedures.

## 1. Introduction

Breast cancer is one of the most common malignancies among women. It is estimated that 37,682 new cases will be diagnosed in Spain by 2025 [1]. This increase in incidence is largely attributed to improvements in early detection techniques such as mammography, tomosynthesis, and magnetic resonance imaging(MRI) [2]. The implementation of systematic screening programs and the use of neoadjuvant therapy have contributed to the identification of non-palpable lesions, improving overall prognosis and expanding the indications for breast-conserving surgery [3,4].

Preoperative localization of these non-palpable lesions is essential to ensure accurate resection and tumor-free margins. Therefore, it is crucial to employ marking techniques that are safe, effective, and do not interfere with imaging or surgical planning [5,6].

Traditionally, metallic wires have been used for localization, although they present significant limitations: they must be placed on the same day as surgery, they may shift, and they often cause discomfort for the patient [7,8]. These drawbacks can compromise surgical accuracy, increase the risk of positive margins, and lead to the removal of larger amounts of breast tissue [9,10]. Nevertheless, their low cost remains a relevant advantage.

Another widely used method is radioguided occult lesion localization (ROLL), which involves the injection of a radioactive tracer followed by intraoperative detection with a gamma probe. Although effective for locating non-palpable lesions [11], ROLL has several limitations, such as the need for a nuclear medicine unit, radiation exposure, short half-life of the radiotracer (6 h), and challenges in cases involving multiple lesions [12,13].

Positive margins are defined as the presence of tumor cells at the edge of the excised tissue, which is associated with a higher risk of residual disease and local recurrence [14]. Margin involvement is defined as tumor on ink for invasive carcinoma, and a minimum surgical margin of 2 mm is established for ductal carcinoma in situ (DCIS) [15].

In response to the limitations of traditional techniques, new technologies such as the SCOUT^®^ (Merit Medical Systems, South Jordan, UT, USA) have emerged. SCOUT^®^ is a non-radioactive radar reflector that can be implanted during diagnostic biopsy, reducing the need for multiple visits to the radiology department. It allows for more flexible surgical planning, provides high localization accuracy, and is compatible with imaging modalities such as MRI [16,17]. Its design eliminates protruding wires, enhancing patient comfort and minimizing the risk of displacement. However, its higher cost and the need for specialized training in order to place the SCOUT^®^ are important considerations.

In response to the limitations of traditional techniques, several wireless localization methods have been developed, including magnetic, radar, and radiofrequency-based systems [16]. Among them, the SCOUT^®^ reflector has shown favourable outcomes in terms of localization accuracy, safety, and MRI compatibility in several institutional series and systematic reviews [17,18,19,20]. Nevertheless, its clinical adoption remains heterogeneous across centres and healthcare systems, and real-world data from Southern European hospitals are still scarce.

This study aims to provide additional real-world evidence on the clinical performance of the SCOUT^®^ localization system for non-palpable breast and axillary lesions, drawing on the experience of a tertiary Spanish university hospital. We report detection success, margin status, reoperation rates, and device-related events, including cases of reflector deactivation—across a consecutive series of 427 patients treated between January 2023 and May 2024.

## 2. Materials and Methods

This is a retrospective observational study carried out at the Hospital Clínico Universitario de Valencia. It included all patients who underwent surgery between January 2023 and May 2024 in which the SCOUT^®^ reflector was used for determining the location of non-palpable breast lesions.

Inclusion criteria were patients scheduled for surgery involving localization of non-palpable breast or axillary lesions using SCOUT^®^ reflector. In the group with malignant pathology, patients diagnosed with breast cancer—both early and advanced stages—were included, some of whom had received neoadjuvant treatment prior to surgery. Patients in whom SCOUT^®^ localization was not performed, or who underwent surgery for reasons unrelated to non-palpable breast lesions, were excluded.

All cases were evaluated by a multidisciplinary breast cancer tumor board, and treatment strategies (neoadjuvant systemic therapy vs. upfront surgery) were defined according to individual case characteristics and current European guidelines. MRI was performed preoperatively in all patients who met one or more of the following criteria: ductal carcinoma in situ (DCIS) with dense breasts (ACR categories C or D), invasive lobular carcinoma, or candidates for neoadjuvant treatment. In patients who underwent neoadjuvant systemic therapy, MRI was systematically repeated after treatment to assess tumor response before surgery.

In cases with a suspicious axillary lymph node, the node was marked with a SCOUT^®^ reflector on the same day the biopsy was performed. Following neoadjuvant therapy, targeted axillary dissection was carried out when a radiological response was observed in the previously affected nodes.

The following main variables were recorded to describe the clinical performance of the technique and surgical outcomes:

- Lesion location: classified according to whether the marker was placed in the axilla or the breast.
- Pathology type: benign or malignant.
- Seed deactivation: any case in which the seed failed to function due to deactivation, including associated complications or difficulties.
- Margin status: tumor-free and positive margins were recorded to evaluate the impact of SCOUT^®^ on surgical precision.
- Reoperations: cases requiring reintervention due to positive margins were documented.
- Neoadjuvant therapy: all patients who received treatment prior to surgery were recorded.
- MRI performed pre- or post-neoadjuvant therapy: whether MRI was performed after neoadjuvant treatment in patients with a SCOUT^®^ already placed.
- MRI after SCOUT^®^ placement: if performed, any imaging artefacts were noted.

## 3. Statistical Analysis

A descriptive cohort analysis was carried out to evaluate the characteristics of qualitative and quantitative variables in the study. Qualitative variables (e.g., malignant/benign status, detection, pathology type) were described using absolute frequencies and percentages.

Quantitative variables (e.g., age) were summarized using measures of central tendency and dispersion: mean and standard deviation (SD) for normally distributed variables, and median and interquartile range (IQR) for those that did not meet this criterion. The normality of quantitative variables was assessed using the Shapiro-Wilk test.

For transparency, 95% confidence intervals (CIs) were calculated for key proportions (using Clopper–Pearson bounds for proportions of 100%).

Statistical analyses were performed using SPSS software, version 26. Descriptive results were presented in tables and graphs to facilitate interpretation.

## 4. Ethics Statement

This study was conducted in accordance with the ethical standards of the institutional research committee and the 1964 Declaration of Helsinki and its later amendments. The study protocol was approved by the Ethics Committee of Hospital Clínico Universitario de Valencia under project number 2025/268. All data were anonymized, and confidentiality was ensured in accordance with current data protection regulations. No additional procedures were performed, and all patients had previously provided informed consent for surgery. Specific informed consent for inclusion in the study was not required due to the retrospective nature of the analysis and the use of anonymized data, in accordance with institutional and national regulations.

## 5. Results

**Figure 1:**
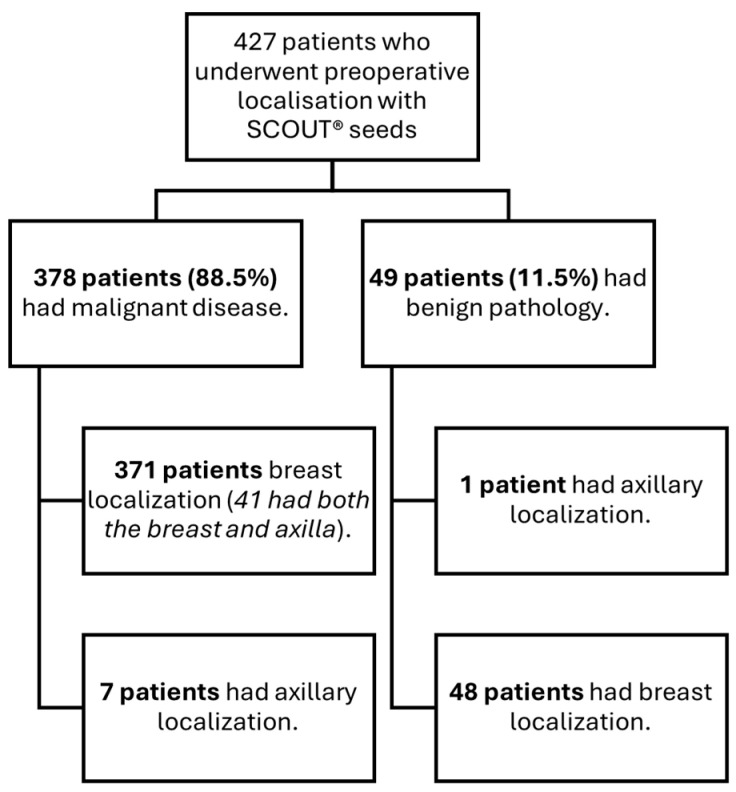
Distribution of patients according to lesion localization and pathological status (malignant vs. benign).

A cohort of 427 patients who underwent surgery between January 2023 and May 2024 was analyzed. All patients had undergone preoperative localization with SCOUT^®^ for one or more non-palpable lesions located in the breast and/or axilla (Figure 1). The mean age of the cohort was 58 ± 12.7 years.

Of the total cohort, 88.5% (*n* = 378) of patients were diagnosed with malignant disease. The mean age in this subgroup was 59.9 ± 11.6 years. In contrast, the benign subgroup (*n* = 49) had a significantly lower mean age of 46 ± 13 years (*p* < 0.001).

Regarding benign lesions localized using the SCOUT^®^, Table 1 outlines the variety of benign pathologies identified, with only one case involving axillary localization in the benign pathology group.

In the malignant group, 92.8% of cases corresponded to invasive carcinoma, 6.1% to carcinoma in situ, and 1.1% to other malignancies. These subtypes are detailed in Table 2, according to molecular profile.

Among patients with malignant disease, 251 (66.4%) underwent MRI at some point during their work-up. Of these, 55 (14.5%) received neoadjuvant systemic treatment prior to surgery. Post-placement MRI was performed in a subset of these patients; only 4 (1.6%; 95% CI: 0.44–4.0) exhibited imaging artifacts attributable to the SCOUT^®^. These artifacts did not compromise staging or surgical planning.

Regarding margin status, 31 patients (8.3%; 95% CI: 5.6–11.4) with malignant pathology presented positive margins. This rate was lower than the previously reported institutional figure of 12%, however, no causal inference can be drawn due to differences in case mix and the lack of a contemporaneous control group. Among these 31 patients, 22 (5.9%; 95% CI: 3.7–8.7) underwent reoperation, consisting primarily of re-excision or mastectomy depending on the extent and nature of margin involvement. Notably, we did not observe a higher rate of positive margins among patients who had received neoadjuvant therapy.

According to institutional criteria, a positive margin is defined as the presence of tumor on ink for cases of invasive carcinoma, and a margin of less than 2 mm for ductal carcinoma in situ (DCIS).

It is important to note that not all patients with positive margins required reintervention. Specifically, those with focally involved ductal carcinoma in situ (DCIS) at the margin were managed conservatively with adjuvant radiotherapy, in accordance with current clinical guidelines and multidisciplinary team recommendations.

Positive margin rates varied according to tumor subtype, Table 3. The highest proportion was observed in in situ carcinoma (26.1%, 95% CI 12.5–46.5). No positive margins were recorded in triple-negative tumors.

Table 4 summarizes the clinical characteristics and comorbidities of patients according to lesion type. No significant differences were observed between groups regarding cardiopathy, diabetes, Chronic Obstructive Pulmonary Disease, smoking status, hypertension, or obesity. The only variable showing a statistically significant difference was neoadjuvant therapy (*p* = 0.017), which, as expected, was exclusively administered to patients with malignant lesions.

All suspicious axillary lymph nodes that had been marked were successfully localized in the operating room (100%; 95% CI: 92.7–100). In contrast, 8 breast localizations (1.9%; 95% CI: 0.8–3.6) failed to be detected intraoperatively. In 5 patients (1.2%; 95% CI: 0.38–2.7), the SCOUT^®^ reflectors were found to be deactivated; however, all of them were localized intraoperatively by manual palpation or ultrasound, Table 5. Notably, most deactivations occurred during the initial training period of a newly incorporated surgeon; however, due to the low number of events, no formal analysis was conducted.

Regarding placement-related events, 10 patients (2.3%) developed a mild haematoma following SCOUT^®^ insertion. No cases of reflector migration, placement difficulty, or need for repositioning were reported. Among the eight cases in which the reflector could not be detected intraoperatively, three were suspected to be related to incorrect placement, possibly due to the seed remaining within the introducing device.

## 6. Discussion

In our study, we evaluated the use of SCOUT^®^ reflectors for the surgical localization of non-palpable breast lesions in 427 patients. Axillary localization was successful in 100% of marked patients, while a non-localization rate of 1.9% (95% CI: 0.8–3.6; 8 patients) was recorded for breast lesions, which is comparable to other series (1.4%) [19]. There were five cases (1.2%) of radar deactivation, three of which were resolved by direct palpation of the seed, and two by ultrasound guidance.

### 6.1. Comparison with Other Methods

When compared to wire localization, SCOUT^®^ reflector has demonstrated lower rates of positive margins (5.6% vs. 13.7%) [16]. Additionally, patients with wire-localized lesions tend to experience higher complication rates, and consequently, require more reinterventions. Although the wire is usually localizable, it may shift or present complications such as wire fracture or haematoma, which may compromise accurate lesion localization and lead to positive margins [20]. A major limitation of wire localization is the requirement for placement hours before surgery, entailing close coordination between the surgical and radiology teams [21]. Nonetheless, its low cost remains a significant advantage.

In comparison, Iodine-125 (I-125) seed localization is associated with a non-localization rate of less than 1% [22]. Its main advantages include preoperative implantation, which facilitates surgical planning [23]. However, it presents drawbacks such as radiation exposure [24] and additional costs associated with need for a dedicated nuclear medicine department, not available in all centres. A study by Zhang et al. [23] published in Annals of Surgical Oncology compared radioactive seed localization (RSL) with wire localization and found that the average cost per patient for RSL was $250.90, compared to $1130.41 for wire localization, including opportunity costs. In our hospital, each seed costs €100, while intraoperative detection costs approximately €150, with an unknown cost attributed to the nuclear medicine physician who supervises the seed from removal to recovery in pathology. The cost of marking a breast lesion with a wire, which involves a prior initial marking session of the lesion with a spherical diagnostic marker, accounts to a combined cost of €252 euros.

Magnetic seed technologies such as Magseed offer the advantage of being radiation-free and can be implanted several days prior to surgery [25]. They are also associated with relatively low rates of positive margins, ranging from 9% to 16.8% [26,27]. Their main drawback is cost, averaging approximately $225 [28]—in our hospital, €450. Although more expensive than other techniques (€2617 for wire localization vs. €2662 for magnetic seed localization), cost-effectiveness is achieved when considering reoperation expenses related to positive margins [29].

### 6.2. Clinical Advantages

Given the variety of localization markers available, it is important to determine the most suitable option for accurate lesion identification while minimizing patient morbidity. However, the ideal marker may vary according to the institution and clinical team. We have implemented the SCOUT^®^ radar system, which enables placement at the time of diagnostic biopsy, avoiding multiple hospital visits for patients. It is suitable for patients undergoing neoadjuvant therapy [16] as it is non-radioactive and can remain indefinitely within the breast. SCOUT^®^ may be particularly valuable in patients who achieve pathological complete response (pCR) after neoadjuvant therapy, as the reflector remains detectable even in the absence of a residual tumor mass, thus facilitating accurate surgical excision or targeted axillary dissection [30]. It is securely anchored, which may reduce the likelihood of displacement and positive margins. In our study, 66.4% of patients underwent MRI, with only four (1.6%; 95% CI: 0.44–4.0) demonstrating minor imaging artefacts.

Our series showed a positive margin rate of 8.3% (CI: 5.6–11.4), which is comparable or even favourable compared to other international reports (5.6–9.5%) [19,21,31,32]. Although this finding is consistent with adequate excision margins, it should be interpreted purely as a descriptive, non-causal association, given the retrospective design and the absence of a contemporaneous control group. The fact that the positive margin rate was lower than the institution’s historical 12% figure should not be interpreted as an improvement, as this difference may simply reflect variations in case mix, surgical technique, or other unmeasured factors rather than any direct effect of SCOUT^®^ use.

Placement-related events were infrequent and mostly minor, consisting of mild haematoma in 2.3% of procedures and two suspected cases of incomplete deployment. No cases of migration or major complications were identified. However, these outcomes were not systematically evaluated and may therefore be underreported.

A notable advantage of the SCOUT^®^ is its MRI compatibility, owing to the absence of ferromagnetic materials in the reflector, which prevents image distortion [17,19]. If the marked lesion is not excised for any reason, the seed can remain safely in place due to its biocompatible materials, which do not provoke adverse tissue reactions [33]. Furthermore, multiple SCOUT^®^ reflectors may be used in the same breast or patient without interference [34].

### 6.3. Device-Related Limitations

SCOUT^®^ is not without its technical limitations. Once deployed by the radiologist, the seed cannot be repositioned, which may be problematic if inaccurately placed [19]. Detection is limited to depths of approximately 6 cm, which can be challenging in large breasts or in the presence of haematomas [29,35]. Deactivation may also occur due to exposure to high temperatures (e.g., from electrocautery) or physical damage such as scissor cuts—although in our experience, functionality may persist even if an antenna is partially severed. Owing to the low number of deactivation cases, we were unable to determine whether a learning curve affects such occurrences. Another practical limitation is the substantial financial investment required. Although SCOUT^®^ is more expensive than wire localization [36], some studies have shown that its precision and ease of use can reduce operative time, optimize scheduling, decrease reoperation rates, and improve patient comfort—potentially offsetting the initial cost [37,38]. However, implementation must be adapted to each institution’s financial and procedural capacity [21]. In our hospital, reflectors are placed at the time of diagnosis, eliminating the need for additional radiology visits and thereby improving workflow efficiency.

### 6.4. Study Limitations

This study also presents methodological constraints inherent to its design. As a retrospective, single-centre observational study, it is subject to potential selection bias, incomplete data capture, and lack of standardized follow-up. The absence of a control group undergoing alternative localization methods limits our ability to establish comparative efficacy or safety. Moreover, confounding factors such as lesion size, multifocality, histological subtype, and the use of neoadjuvant therapy were not controlled and may have influenced surgical outcomes.

These limitations mean that the observed findings—such as the lower rate of positive margins—should be interpreted as descriptive, non-causal associations rather than evidence of improved efficacy attributable to SCOUT^®^. The single-institution context may also limit extrapolation to other clinical settings.

This study focuses primarily on intraoperative and immediate postoperative endpoints. Long-term oncologic safety, including local recurrence and disease-free survival, cannot be assessed based on the available data. Therefore, the present results should be interpreted as preliminary but promising, warranting confirmation in prospective studies with extended follow-up.

### 6.5. Future Directions

The flexibility of SCOUT^®^ placement at any point prior to surgery enhances scheduling for both patients and clinical teams, reduces operating room delays, improves hospital logistics, and alleviates patient anxiety [31]. Additionally, the impact on patient comfort warrants further evaluation, as performing lesion localization on the same day as the diagnostic biopsy spares patients an additional hospital visit.

Future prospective, multicentre studies with standardized data collection and longer follow-up are warranted to validate these observations and better assess the oncologic and procedural impact of SCOUT^®^-guided localization.

## 7. Conclusions

Given the growing emphasis on breast-conserving surgery and patient-centered care, technologies such as SCOUT^®^ represent a promising tool to support a more precise and less invasive surgical approach to breast cancer.

In our experience, SCOUT^®^-guided localization of non-palpable breast lesions was associated with accurate lesion excision and a low rate of positive margins, thereby reducing the need for reintervention.

Although a few cases of non-localization and deactivation were observed, these were infrequent and effectively managed with intraoperative rescue techniques, supporting the clinical feasibility of the method.

While its cost is higher than that of traditional localization systems, its advantages in workflow flexibility, surgical precision, and patient comfort make SCOUT^®^ a valuable option in the context of breast-conserving surgery.

Nevertheless, given the retrospective, single-center design of this study, these results should be interpreted as descriptive and hypothesis-generating, warranting confirmation in prospective multicenter studies with long-term follow-up.

## Figures and Tables

**Table 1:** Description of benign pathology cases.

		*n*	%
**Breast**	Fibroadenoma	20	40.8
	Atypical Ductal Hyperplasia	12	24
	Sclerosing Adenosis	1	2
	Papilloma	11	22.4
	Hamartoma	3	6.1
	Lobular Carcinoma In Situ	1	2
**Axilla**		1	2

**Table 2:** Description of malignant pathology cases.

	*n*	%
*Luminal A*	153	40.5
*Luminal B*	117	31
*Luminal B + Her2+*	16	4.2
*Her2+*	23	6.1
*Triple—Negative*	41	10.8
*Carcinoma* In Situ	23	6.1
*Papillary Carcinoma*	1	0.3
*Sarcoma*	1	0.3
*Lymphoma*	2	0.5
*Urothelial Metastasis*	1	0.3

* HER2 (Human Epidermal Growth Factor Receptor 2).

**Table 3:** Positive Margin Rates by Tumor Subtype.

Subtype	*n*	Positive Margin	% Positive Margin
**Luminal A**	153	14	9.2
**Luminal B**	117	8	6.8
**Luminal B HER2+**	16	1	6.3
**HER2+**	23	1	4.3
**Triple negative**	41	0	0.0
**In situ (DCIS)**	23	6	26.1
**Other**	5	1	20.0

**Table 4:** Clinical characteristics and comorbidities of patients according to lesion type.

Variable	Total %	Malignant %	Benign %	*p*-Value
**Cardiopathy**	3.4	4.3	0.0	1.000
**Diabetes**	13.2	11.9	0.0	0.600
**Chronic Obstructive Pulmonary Disease**	4.7	4.3	0.0	1.000
**Smoker**	15.1	15.9	0.0	0.353
**Hypertension**	20.7	20.5	0.0	0.206
**Obesity**	24.7	28.2	20.0	0.726
**Neoadjuvant therapy**	32.5	34.5	0.0	0.017

**Table 5:** Summary of SCOUT^®^ deactivation events successfully managed intraoperatively.

Month of Deactivation	Method of Intraoperative Localization	Outcome
*02 - 2023*	Ultrasound	Successful excision
*03 - 2023*	Palpation	Successful excision
*03 - 2023*	Palpation	Successful excision
*04 - 2023*	Ultrasound	Successful excision
*10 - 2024*	Palpation	Successful excision

## Data Availability

The data presented in this study are available on reasonable request from the corresponding author. The data are not publicly available due to privacy and ethical restrictions.
